# The role of Tumor necrosis factor alpha −308 G>A promoter polymorphism in pediatric community acquired pneumonia

**DOI:** 10.1186/s43054-020-0019-1

**Published:** 2020-02-10

**Authors:** Fady M. El Gendy, Muhammad Said El-Mekkawy, Sherin Sobhy El-Naidany, Shimaa Tarek El-torgoman

**Affiliations:** 1 Department of Pediatrics, Faculty of Medicine, Menoufia University, Menoufia, 32511 Egypt; 2 Department of Biochemistry, Faculty of Medicine, Menoufia University, Menoufia, 32511 Egypt

**Keywords:** Tumor necrosis factor alpha, Pneumonia, Pediatric, Polymorphism, −308 G>A polymorphism

## Abstract

**Background:**

Tumor necrosis factor alpha (TNF-α) −308 G>A promoter polymorphism might be associated with excessive production of the proinflammatory cytokine TNF-α, modulating host response to pulmonary infections. Our objective was to evaluate the association of TNF-α gene −308 G>A polymorphism with susceptibility to, and severity of, community-acquired pneumonia (CAP).

**Results:**

This was a cross-sectional study including 45 Egyptian children hospitalized for CAP in addition to 45 healthy children who served as a control group. Pneumonia severity was assessed on admission by the World Health Organization (WHO) guidelines; Pediatric Respiratory Severity Score (PRESS) score; Predisposition, Infection, Response and Organ failure (PIROm) score; and Respiratory Index of Severity in Children (RISC) score. Genotyping of TNF-α polymorphism was performed to all individuals by polymerase chain reaction and restriction fragment length polymorphism (PCR-RFLP). Patients were monitored till hospital discharge. Frequency of AG genotype was lower among patients compared with control [odds ratio (OR) and 95% confidence interval (CI) = 0.13 (0.03–0.63); *p* = 0.012]. Prevalence of genotypes AA+AG was lower among patients compared with controls [OR and 95% CI = 0.34 (0.12–0.99); *p* = 0,048]. The “A” allele prevalence was higher among controls, but no significant association was found with CAP [OR and 95% CI = 0.58 (0.25–1.35); *p* = 0.21]. When PRESS score was used to classify patients into “severe pneumonia” and “non-severe pneumonia,” no significant association of any of the alleles or genotypes with CAP severity was found.

**Conclusion:**

TNF-α −308 G>A polymorphism confers protection from pediatric CAP but is not associated with indicators of CAP severity. Larger studies are needed to confirm these findings in pediatric patients from different ethnicities.

## Background

Pediatric community-acquired pneumonia (CAP) is defined as the presence of signs and symptoms of pneumonia in a previously healthy child due to an infection which has been acquired outside hospital [1]. Despite marked decrease in childhood mortality and pneumonia-specific mortality, CAP remains the major cause of mortality in younger children globally, causing about 900,000 child deaths in 2013 [1].

In response to CAP, an inflammatory reaction is produced locally in the lung which consists of both pro-inflammatory and anti-inflammatory cytokines, including interleukins (IL), granulocyte colony-stimulating factor (G-CSF), granulocyte-macrophage colony-stimulating factor (GM-CSF), and tumor necrosis factor alpha (TNF-α) [2].

TNF-α is potent pleiotropic pro-inflammatory cytokine produced mainly by activated macrophages, lymphocytes, and endothelial cells [3]. Human TNF-α is a 17-kDa protein that exists in a soluble form or a membrane-bound form. TNF-α gene is located within the major histocompatibility complex (MHC) on the short arm of chromosome 6 [4].

Historically, the name “tumor necrosis factor” referred to a “factor” induced by bacterial infections that leads to tumor regression [5]. It was later discovered that TNF-α has a wide range of biological effects on host defense against pathogenic agents. It can induce cell survival, proliferation, and differentiation. It can also cause both apoptosis and necrosis under certain conditions [6].

TNF-α function is double-faceted. On the one hand, local production of TNF-α is beneficial in the acute situation since it increases expression of adhesion molecules on the vascular endothelial cells that help immune cells to migrate to sites of infection [4]. Moreover, TNF-α activates phagocytes to engulf infectious agents. On the other hand, systemic or prolonged elevation of TNF-α level may be harmful. High level of circulating TNF-α is associated with toxic shock induced by endotoxins [7]. TNF-α injection into experimental animals causes a syndrome similar to septic shock [8] and infusion of TNF-α into humans results in systemic inflammatory response syndrome [9]. TNF-α and IL-1 act synergistically to induce a shock-like state characterized by increased vascular permeability, pulmonary edema, and hemorrhage [10].

In addition, TNF-α was found to play a major role in the pathogenesis of inflammatory diseases like rheumatoid arthritis, ankylosing spondylitis, psoriatic arthritis, Behçet’s disease, and inflammatory bowel diseases [11].

A large number of polymorphisms of TNF-α gene promoter have been described which are thought to affect production of TNF-α. One of these single-nucleotide polymorphism (SNP) occur at nucleotide number 308 before the transcription start site where nucleotide “G” is changed to “A” (−308 G>A). This SNP could potentially affect TNF-α synthesis at the transcriptional level. The “A” allele was suggested to be associated with higher TNF-α levels which may alter the course of immune response [12].

Although fairly well evaluated in adult CAP patients, previous pediatric literature evaluating the role of TNF-α −308 G>A polymorphism in CAP is scarce. The aim of the present study was to test the hypothesis that TNF-α −308 G>A polymorphism is a risk factor for susceptibility to CAP and for having a severe disease course.

## Methods

This was a cross-sectional study conducted on 90 Egyptian children from September 2018 to April 2019. The local ethical committee approved the study protocol. The study population included a group of 45 children with a diagnosis of CAP who were consecutively enrolled from the ward and the pediatric intensive care unit (PICU) of a university hospital after obtaining a written informed consent from the parents. Another group of 45 age- and sex-matched healthy children served as a control group.

Children from the age of 2 months to 5 years hospitalized with a diagnosis of CAP were eligible for inclusion in the study after obtaining an informed parental consent. We specifically chose this age range since it is the one included in the World Health Organization (WHO) classification of pneumonia severity which was the main classification used in the present study for the purpose of evaluating the relation of TNF-α polymorphism to CAP severity. The exclusion criteria were (1) children with known immunodeficiency or taking immunosuppressive drugs, (2) suspected tuberculosis, (3) chronic respiratory disorders, (4) coexistence of another infection with CAP, e.g., gastroenteritis, (5) persistent asthma, (6) severe malnutrition, and (7) a pre-existent cardiac disease. In addition, children were excluded from the control group if the parents gave a history of previous CAP episode.

CAP was diagnosed in the presence of signs and symptoms of acute lower respiratory infection that has been acquired outside the hospital and that was confirmed by demonstration of an infiltrate on chest X-ray [13].

Patients were evaluated through full history, general examination, and local examination of the chest to detect signs of lower respiratory tract infection and those indicating respiratory distress, e.g., chest indrawing. Moreover, pneumonia severity was assessed by some clinical scoring systems, namely the Pediatric Respiratory Severity Score (PRESS) score [14]; Predisposition, Infection, Response and Organ failure (PIROm) score [15]; Respiratory Index of Severity in Children (RISC) score, and the WHO classification [16]. Laboratory investigations included complete blood count (CBC), C-reactive protein (CRP), blood gas analysis, serum electrolytes, and renal function tests. Blood culture was performed for patients needing PICU admission.

In addition, analysis of TNF-α −308 G>A promoter polymorphism was performed to all patients and controls by polymerase chain reaction followed by restriction fragment length polymorphism (PCR-RFLP) as previously described [17]:-

### DNA extraction

A 2-mL venous blood sample was drawn from all study participants and stored in EDTA tubes.

Genomic DNA was extracted from peripheral blood using a using Thermo Scientific Gene JET Genomic DNA purification kit (Lithuania). DNA was eluted and stored at − 20 °C for further PCR procedure.

### Polymerase chain reaction (PCR)

A 116 bp DNA fragment was amplified, using the forward primer 5′-AGG CAA TAG GTT TTG AGG GCC AT-3′ and the reverse primer 5′-ACA CTC CCC ATC CTC CCT GCT-3′. PCR amplification was performed by Applied Bio systems 2720 thermal cycler (Singapore). The amplification reaction contained 200 ng of template DNA, 1 μL of each primer (20 mM each), 25 μL of PCR master mix (My Taq Red Mix, 2x, Bioline Reagents Ltd, London, UK), and water (ddH2O) up to 50 μL.

The PCR reaction involved the following steps: an initial denaturation of 95 °C for 2 min then 35 cycles of 95 °C for 30 s; 60 °C for 15 s; 74 °C for 15 s; and a final extension step at 74 °C for 10 min.

### Restriction fragment length polymorphism

The amplified PCR product was cleaved by NcoI restriction enzyme (10 U/μL; Thermo Fisher Scientific; Waltham, MA, USA) where1 μL of NcoI was added to 10 μL PCR reaction mixture, 18 μL nuclease free water, and 2 μL 10X Buffer Tango. The mixture was gently mixed and spun for a few seconds then incubated at 37 °C for 16 h then subjected to agarose gel (3%) electrophoresis and visualized by ethidium bromide staining and UV transillumination. A 100-bp DNA ladder (BioLabsInc, New England) was used. The presence of a single band at 116 bp indicated AA homozygous genotype. The presence of two bands at 96 and 20 bp indicated GG homozygous genotype, and finding of three bands at 116, 96, and 20 bp indicated AG heterozygous genotype.

### Genetic model analysis

We used three genetic models, namely “genotype/co-dominant,” “recessive,” and “dominant” models to test the association of −308 TNF-α polymorphism with CAP. In the genotype/co-dominant model, the frequencies of each of the different genotypes (GG vs AA vs AG) among patients and controls were compared. In the recessive model, the frequency of AA genotype was compared versus that of AG+GG among patients and controls. In the dominant model, the frequency of AA+AG genotypes was compared versus GG.

### Statistical analysis

Qualitative data was expressed as number (%) and analyzed using a chi-square test. Normally distributed continuous variables were expressed as the mean ± standard deviation while continuous variables with non-normal distribution were presented as the median (minimum–maximum). Normally distributed continuous variables were compared using a *t* test while non-normally distributed continuous variables were compared using the Mann-Whitney *U* test (for two variables) or the Kruskal-Wallis test (for more than two variables). Logistic regression analysis was used to test the association of different alleles and genotypes with CAP. This yielded odds ratio (OR) and 95% confidence interval (95% CI). If the odds ratio is > 1, this means positive association (the variable increases the risk of occurrence of an event). If the odds ratio < 1, this indicates a negative association (the variable decreases the risk of occurrence of an event). All statistical analyses were performed using IBM SPSS software version 23.0 (SPSS, Inc., Chicago, IL, USA). A *p* value < 0.05 was considered statistically significant.

## Results

### Characteristics of the study population

Forty-five patients and 45 controls were enrolled. Their basic characteristics are shown in Table 1. 37.8% of patients had previous one or more episodes of pneumonia. Sixty percent had lobar consolidation; the remaining had patchy or interstitial infiltrate. The median PIROM, PRESS, and RISC scores were 0, 3, and 2 respectively. Only three children (6.6%) had severe pneumonia under the WHO classification. Of note, none of our patient cohort died.

**Table 1:** Demographic, clinical, and laboratory characteristics of patients

Variable	Patients (*n* = 45)	Controls (*n* = 45)	*p* value
Age, months	19 (2–60)	20 (1.5–60)	0.68
Male sex	32 (71.1%)	24 (53.3%)	0.52
Weight, kg	11.8 ± 4.1	13.4 ± 5.2	0.23
Height , cm	78 ± 14.5	85 ± 15.7	0.45
Malnutrition	2 (4.4%)	NA	NA
Prematurity	2 (4.4%)	3 (6.6%)	0.64
Indoor smoking	28 (62%)	23 (51.1%)	0.28
Exclusive breastfeeding	33 (73%)	27 (60%)	0.17
Previous pneumonia	17 (37.8%)	0 (0%)	< 0.001*
Temperature, °C	38 ( 37–39)	37 (36.8–37.4)	< 0.001*
RR/min	45 ( 30–72)	31 (23–40)	< 0.001*
SpO_2_ in room air	97% (95–99%)	NA	NA
ARDS	0 (0%)	NA	NA
Shock	0 (0%)	NA	NA
Invasive MV	0 (0%)	NA	NA
Lobar consolidation	27 (60%)	NA	NA
Pleural effusion	0 (0%)	NA	NA
Length of hospital stay, days	6 (3–10)	NA	NA
PICU admission	3 (6.6%)	NA	NA
Mortality	0 (0%)	NA	NA
PIROm score	0 (0–1)	NA	NA
PRESS score	3 (1–5)	NA	NA
RISC score	2 (0–3)	NA	NA
WHO classification			
Pneumonia	42 (93.3%)	NA	NA
Severe pneumonia	3 (6.6%)		
WBC (1000/μL)	9.05 (4.2–21)	NA	NA
Hemoglobin, g/dL	11.3 ± 1.2	NA	NA
Platelets (1000/μL)	340.8 ± 86.6	NA	NA
CRP, mg/dL	24 (0–96)	NA	NA

Data is expressed as the mean ± SD, median (minimum–maximum), or number (percentage)

*RR* respiratory rate, *SpO*_*2*_ saturation of peripheral oxygen, *ARDS* acute respiratory distress syndrome, *MV* mechanical ventilation, *PICU* pediatric intensive care unit, *PIROm* Predisposition, Insult, Response, Organ dysfunction modified score, *PRESS* Pediatric Respiratory Severity Score, *RISC* Respiratory Index of Severity Score, *WBC* white blood cell count, *CRP* C-reactive protein

*Statistically significant

### Allele and genotype distribution among patients and controls

The frequencies of different alleles and genotypes were compared among patients and controls under the recessive, dominant, and genotype models (Table 2).

**Table 2:** Distributions of the TNF-α genotypes and alleles among patients and controls

	Patients (*n* = 45)	Control (*n* = 45)	OR (95% CI)	*p* value
Genotype model				
AA	4 (8.9%)	2 (4.4%)	1.6 (0.27–9.25)	0.61 0.012^a^
AG	2 (4.4%)	12 (26.7%)	0.13 (0.03–0.63)	
GG	39 (86.7%)	31 (68.9%)	Reference	
Dominant model				
GG	39 (86.7%)	31 (68.9%)	Reference	0.048^a^
AA+AG	6 (13.3%)	14 (31.1%)	0.34 (0.12–0.99)	
Recessive model				
AA	4 (8.9%)	2 (4.4%)	2.09 (0.36–12.07)	0.4
AG+GG	41 (91.1%)	43 (95.6%)	Reference	
Alleles				
A	10 (11.1%)	16 (17.7%)	0.58 (0.25–1.35)	0.21
G	80 (88.8%)	74 (82.2%)	Reference	

Data is expressed a number (percentage)

*OR* odds ratio, *95% CI* confidence interval

^a^Statistically significant

Under the genotype model, the AG genotype frequency was significantly lower among patients compared with control. The odds ratio was< 1 [OR and 95% CI = 0.13 (0.03–0.63); *p* = 0.012], implying that the association was negative, that is, AG genotype decreases the risk of acquiring CAP.

Under the dominant model, the frequency of the pooled genotypes AA+AG was significantly lower among patients compared with controls. The OR and 95% CI were 0.34 (0.12–0.99) and *p* = 0.048, implying that the presence of either AA or AG significantly reduced susceptibility to CAP.

Under the recessive model, no significant difference was found in the prevalence of AA genotype compared with GG+AG genotypes [OR = 2.09 (0.36-12.07); *p* = 0.4]

The frequency of “A” allele was lower among patients but no significant association was found with CAP [OR and 95% CI = 0.58 (0.25–1.35); *p* = 0.21]

### The relation of genotypes and alleles to CAP severity

When PRESS score was used to classify patients into “severe pneumonia” and “non-severe pneumonia,” no significant association of any of the alleles or genotypes with CAP severity was found under the dominant, recessive, or genotype models (Table 3).

**Table 3:** The association of genotypes and alleles with pneumonia severity

	Severe pneumonia (*n* = 9)	Non-severe pneumonia (*n* = 36)	*p* value
Genotype			
GG	7 (77.8%)	32 (88.9%)	0.52
AG	1 (11.1%)	1 (2.8%)	
AA	1 (11.1%)	3 (8.3%)	
Dominant model			
AA+AG	2 (22.2%)	4 (11.1%)	0.38
GG	7 (77.8%)	32 (88.9%)	
Recessive model			
GG+AG	8 (88.9%)	33 (91.7%)	0.79
AA	1 (11.1%)	3 (8.3%)	
Allele			
A	3 (16.6%)	7 (9.7%)	0.41
G	15 (83.3%)	64 (90.2%)	

Severity according to PRES score, data is expressed as number (%)

The median PRESS, RISC, and PIROm scores tended to be lower among patients with AA genotype, but the difference was not significant (Table 4).

**Table 4:** The effect of genotype on pneumonia severity scores

	Genotype			*p* value
	AA (*n* = 4)	AG (*n* = 2)	GG (*n* = 39)	
PRESS	2 (2–3)	3.5 (3–4)	3 (1–5)	0.44
PIROm	0 (0–1)	0 (0–0)	0 (0–1)	0.74
RISC	1 (1–1)	1.5 (1–2)	2 (0–3)	0.23

Data is expressed as median (minimum–maximum)

## Discussion

Scientists have been increasingly aware that the invading pathogens are not entirely to blame for the severity of infectious processes since the excessive inflammation produced by the host plays a significant role.

In the present study, we evaluated the role of one polymorphism in the promoter of TNF-α gene. Our main finding was that the frequency of AG genotype and that of the AA+AG genotypes were significantly lower among CAP patients compared with controls and logistic regression analysis indicated a significant negative association of genotypes with CAP. The prevalence of “A” allele was also lower among patients, but the difference was not statistically significant.

Together, these findings could be construed as implying that TNF-α G>A promoter polymorphism confers protection from CAP development. Surprisingly, our findings came against the hypothesis that more TNF production by the “A” allele increases susceptibility to CAP. However, it is also reasonable to suggest a counter hypothesis: more TNF-α production ensures more complete pathogen eradication. This could explain the finding reported by a study of patients with influenza pandemic (H1N1) wherein the frequencies of “A” allele and GA genotype were significantly lower while those of the G allele and GG genotype were significantly higher among patients compared with controls [18]. Similarly, another study reported that the patients with influenza-related pneumonia were more frequently homozygous for the G allele of the TNF 308 G/A polymorphism compared with controls [19].

Notwithstanding, one should be cautious while drawing firm conclusions from our current research due to the small sample size. Moreover, the inflammatory reaction requires the cooperation of many different genes. The small effect of each gene requires a large sample size to be elicited in association studies. Furthermore, −308 G>A polymorphism is not the only polymorphism inside TNF-α promoter; other polymorphisms include −1031 (T/C), −863 (C/A), −857 (C/A), −851 (C/T), −419 (G/C), −376 (G/A), −238 (G/A), −162 (G/A), and −49 (G/A) [12]. Consequently, the level of TNF-α is likely the product of several of these polymorphisms and not just −308 G>A [20], necessitating a thorough evaluation of all polymorphisms in future larger studies.

It should be pointed out that, to the best of our knowledge, our current study is the first to evaluate the role of −308 G>A polymorphism in pediatric CAP patients, but a relevant study of 120 very low birth weight mechanically ventilated infants found that the incidence of nosocomial pneumonia was not significantly different between infants with GG genotype and those having AA/AG [21].

In another study of 69 adult patients with pneumococcal disease (61 with CAP, 5 with meningitis, and 3 having both), no significant difference in the distribution of TNF-α was found between patients and controls [22]. Likewise, a large multicenter study found no significant difference in the distribution of alleles or genotypes of −308 G>A polymorphism between adult CAP patients and controls [23]. Similarly, a meta-analysis of 12 studies (the great majority were adult studies) concluded that −308 G>A polymorphism (AA+AG vs GG) was not associated with CAP or hospital-acquired pneumonia (HAP) risk, but when subgroup analysis was performed, the polymorphism was found to be associated with pneumonia in Asians but not in Caucasians [24].

It seems that the latter multicenter study and meta-analysis somewhat discouraged researchers from pursuing further research on the issue. However, it should be noted that the situation in pediatric patients is far from clear and the influence of ethnicity needs a more thorough evaluation.

In addition to the effect of TNF-α polymorphism on CAP susceptibility, we evaluated a potential influence of the polymorphism on CAP severity. We found no significant association of alleles or genotypes with any indicators of CAP severity, including clinical severity scores, but we were not able to assess the influence of TNF-α polymorphism on mortality since none of our patients died. It is likely that the small sample size could be responsible for our failure to demonstrate an association of TNF-α with any indicators of CAP severity. However, previous studies generally point to lack of association of this polymorphism with CAP outcome.

Consistent with our findings, genotyping of 77 children with respiratory syncytial virus infection revealed no association of TNF-α −308 G>A polymorphism with any of the clinical outcomes, including severity scores of lower respiratory illness, oxygen saturation, lengths of oxygen supplementation, intensive care unit (ICU) and hospital stays, and the presence or absence of pneumonia and otitis media [25]. However, another study of 277 Chinese adult patients with severe pneumonia-induced sepsis found that TNF-α “A” allele increased the risk of septic shock (OR = 4.28). Moreover, the combined GA+AA increased the risk of septic shock even after adjustment for confounding factors (OR = 2.96) However, no association of alleles or genotypes was found with mortality [26].

In a multicenter study, no significant association of allele or genotype of −308 G>A polymorphism with adult CAP severity and outcome was noted [23]. Similarly, a meta-analysis concluded that genotype (AA+AG) was not associated with a higher risk of mortality from pneumonia compared with GG, but carriers of “A” allele had higher risk of having severe pneumonia [24].

The unexpected lack of association of TNF-α −308 G>A polymorphism with CAP outcome might be explained by the presence of additional factors which cooperate together to determine the level of inflammatory response which, in turn, is presumed to influence the final prognosis. These factors include the following: first, variations in the levels of pro-inflammatory cytokines other than TNF-α; second, variation in the level of TNF-α from the influence of other promoter polymorphisms; third, variations in the responses of different individuals to the same TNF-α level due to polymorphisms in TNF-α receptor genes or in the genes encoding signal transduction pathway molecules; fourth, the balance between pro-inflammatory and anti-inflammatory genes; fifth, the inflammatory response to CAP in children might be somewhat different from that in adults; and sixth, the pattern of cytokine response might vary according to the pathogen type since different organisms interact with different pattern recognition receptors (PRR), with different signaling pathways [27].

So, it should be born in mind that if the excessive inflammatory response is associated with severe CAP, it is not TNF-α alone which determines the level of this inflammation, and some patients may demonstrate excessive TNF-α gene expression but its effects become overwhelmed by the other factors. Undoubtedly, a large association study or a meta-analysis of a greater number of studies is needed for more clarification of the issue.

The main limitation of the present study is the small sample size. Another limitation is the lack of measurement of serum TNF-α level to evaluate its relation to different alleles and genotypes. In addition, patients with severe pneumonia were a minority among our patient cohort and it is possible that our findings could have been different if more patients had severe pneumonia. These limitations need to be avoided in future studies.

## Conclusion

In conclusion, TNF-α −308 G>A polymorphism appears to confer protection from pediatric CAP among Egyptian children but it is not associated with indicators of CAP severity. Larger studies in different populations are needed to confirm the role of this polymorphism in pediatric CAP.

## Data Availability

Not applicable

## Abbreviations

CAP: Community-acquired pneumonia
CBC: Complete blood count
CI: Confidence interval
CRP: C- reactive protein
G-CSF: Granulocyte colony-stimulating factor
GM-CSF: Granulocyte-macrophage colony-stimulating factor
HAP: Hospital-acquired pneumonia
IL-1: Interleukin-1
MHC: Major histocompatibility complex
OR: Odds ratio
PCR: Polymerase chain reaction
PICU: Pediatric intensive care unit
PIROm: Predisposition, Infection, Response and Organ failure (PIROm) score
PRESS: Pediatric Respiratory Severity Score
RFLP: Restriction fragment length polymorphism
RISC: Respiratory Index of Severity in Children
SNP: Single-nucleotide polymorphism
TNF-α: Tumor necrosis factor alpha
WHO: World Health Organization
